# Multiparametric US and MRI Features of Femoral Myxoid Liposarcoma—Case Report and Literature Review

**DOI:** 10.3390/diagnostics16091286

**Published:** 2026-04-24

**Authors:** Thomas Ferenc, Nikolina Jurjević, Andro Matković, Lea Korša, Kristian Kunjko, Ana Terezija Jerbić Radetić, Ivana Jurca, Ranko Smiljanić, Helga Sertić Milić, Vinko Vidjak

**Affiliations:** 1 Department of Diagnostic and Interventional Radiology, Merkur University Hospital, 10000 Zagreb, Croatia; nikolinajurjevic@yahoo.com (N.J.); rtg.smiljanic@gmail.com (R.S.); vinko.vidjak@gmail.com (V.V.); 2 Department of Pathology and Cytology, University Hospital Centre Zagreb, Kispaticeva 12, 10000 Zagreb, Croatia; lea.korsa@gmail.com; 3 Department of Plastic and Reconstructive Surgery and Breast Surgery, University Hospital Centre Zagreb, Kispaticeva 12, 10000 Zagreb, Croatia; kkunjko@kbc-zagreb.hr (K.K.); ana.jerbic.radetic@uniri.hr (A.T.J.R.); 4 School of Medicine, University of Zagreb, 10000 Zagreb, Croatia; 5 Department of Anatomy, Medical Faculty, University of Rijeka, 51000 Rijeka, Croatia; 6 Department of Diagnostic and Interventional Radiology, University Hospital Centre Zagreb, Kispaticeva 12, 10000 Zagreb, Croatia

**Keywords:** ultrasound, contrast-enhanced ultrasound, strain elastography, B-flow imaging, magnetic resonance imaging, liposarcoma

## Abstract

**Background/Objectives**: Myxoid liposarcoma (MLS) is a malignant soft-tissue tumor and the second-most common subtype of liposarcoma, often occurring in the lower limbs of middle-aged patients. **Case Presentation**: A 38-year-old male patient presented to the ultrasound outpatient clinic with a large mass in the right femoral region. It has been present for 15 years and mostly stable in size. Over the last two years, it has been slowly increasing in size, more rapidly in the previous 10 months, and has started to limit his range of motion. After multiparametric ultrasound and magnetic resonance imaging evaluation, the proposed diagnosis was myxoid liposarcoma. Following imaging workup, the patient was referred to the tertiary sarcoma center, where a biopsy was performed, and pathohistological diagnosis was low-grade myxoid liposarcoma. Contrast-enhanced computed tomography (CT) evaluation of the thorax, abdomen, and pelvis showed no signs of dissemination, and CT angiography showed no signs of vessel infiltration. Plastic surgery and vascular surgery specialists performed the extirpation of the mass with the partial resection of the adjacent sartorius muscle and the complete resection of the great saphenous vein. Subsequent pathohistological analysis of the mass and local lymph nodes showed clear surgical margins and no lymphatic or vascular invasion. The patient is currently under regular surveillance by an oncology specialist and awaiting adjuvant radiotherapy. **Conclusions**: A multidisciplinary approach is essential in the management of patients with MLS, as it provides a tailored, individualized assessment from diagnosis through treatment to ensure the best possible outcome.

## 1. Introduction

Myxoid liposarcoma (MLS) is a malignant soft-tissue tumor that accounts for 20–50% of liposarcoma cases [1,2]. It is usually found in the lower limb (75–97.5% of cases) and in patients aged 43–48 years, a decade earlier than in other liposarcoma subtypes [2,3]. MLS is characterized by variable round-cell content, with a higher proportion in more aggressive tumors [2]. It is divided into low-grade and high-grade subtypes. Low-grade MLS is usually a slow-growing mass with a low risk of metastases. In contrast, high-grade MLS (>5% round cell morphology) is associated with a higher risk of metastases and a poor survival rate [1]. Multiparametric ultrasound (MPUS) is usually the first imaging modality for evaluating soft-tissue masses, and magnetic resonance imaging (MRI) is the mainstay for preoperative assessment [4,5,6]. Herein, we report a case of a male patient with femoral low-grade MLS with emphasis on imaging assessment.

## 2. Case Report

A 38-year-old male patient presented to the ultrasound outpatient clinic with a large mass in the right femoral region. It has been present for 15 years, predominantly small and stable in size. The patient was not referred for diagnostic workup because the mass was considered to be a subcutaneous lipoma. However, over the last two years, it has slowly increased in size, more rapidly over the past 10 months, and has begun to limit his range of motion. The patient denied any trauma or recent surgery of the right femoral region. His past medical history was remarkable for multiple body lipomas (like his father), right renal agenesis, and surgical intervention for left ureteropelvic junction obstruction at the age of 3; otherwise, he was a healthy individual. During physical examination, there was no skin discoloration or wounds of the right femoral region, and the mass was fixed, soft, and painless at palpation.

**Figure 1:**
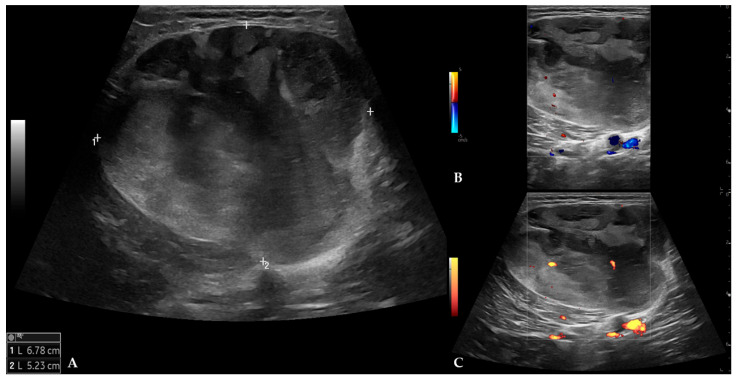
B-mode, color, and power Doppler images of MLS: (**A**) On B-mode, MLS presented as a heterogeneous mass with hypoechoic foci; (**B**) on color Doppler, MLS was poorly vascularized with a low arterial peak systolic velocity of 13 cm/s (not shown on image); (**C**) on power Doppler, MLS also demonstrated mild central vascularity.

**Figure 2:**
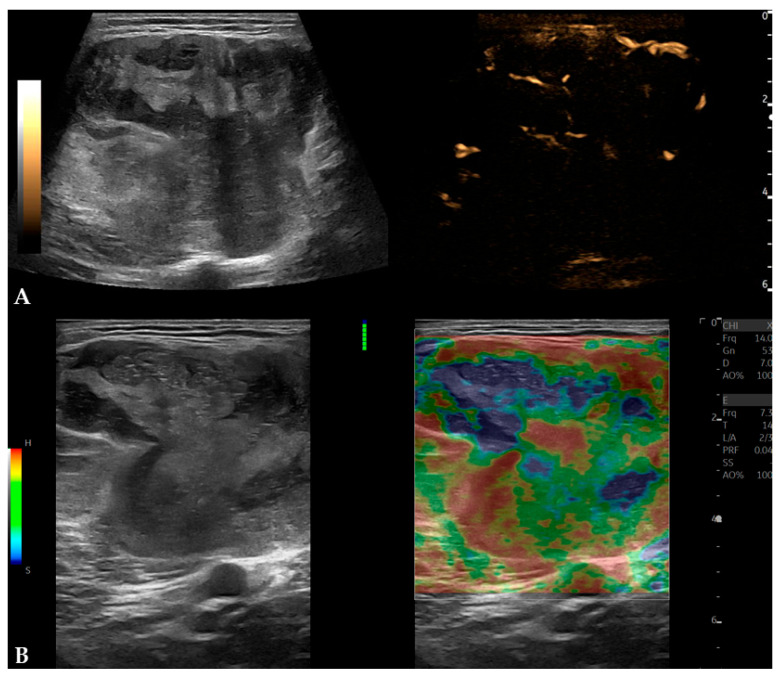
B-flow imaging and strain elastography of MLS: (**A**) B-flow imaging showed MLS to be moderately vascularized; (**B**) strain elastography showed MLS to be predominantly a soft mass, resembling an elastography score of 1 according to Griffith [6], which is a characteristic for benign masses (strain ratio and E/B ratio were not measured due to technical limitations and 2D SWE was not available at the time of examination).

MPUS examination (Logiq Fortis, GE Healthcare, Chicago, IL, USA) demonstrated a well-marginated, expansive mass measuring 6.8 × 5.2 × 9.1 cm, with heterogeneous echogenicity and no signs of infiltration into adjacent structures. On color and power Doppler evaluation, the mass was poorly vascularized with low-flow velocity arteries and veins (peak systolic velocity of arterial flow was 13 cm/s) (Figure 1). B-flow imaging showed that the mass was moderately vascularized with low-flow blood vessels. On strain elastography, the mass demonstrated heterogeneous stiffness but was predominantly soft (2D shear-wave elastography (2D SWE) was not available at the time of examination) (Figure 2).

**Figure 3:**
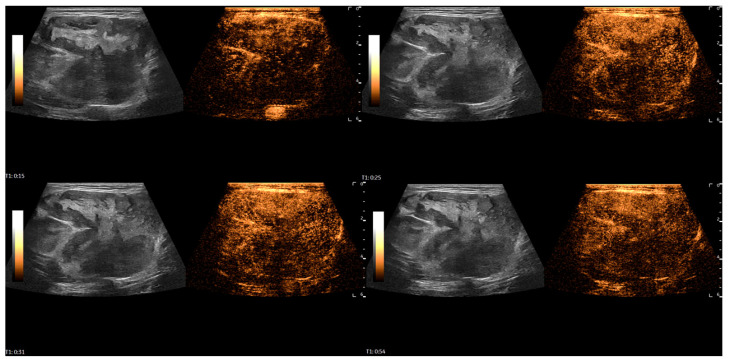
A series of CEUS images demonstrating heterogeneous enhancement of the MLS resembling the P3 pattern (according to Loizides et al. [7]) or the P6 pattern of perfusion (according to De Marchi et al. [8]). Enhancement started approximately 15 s into scanning. It raised suspicion of a malignant soft-tissue mass.

**Figure 4:**
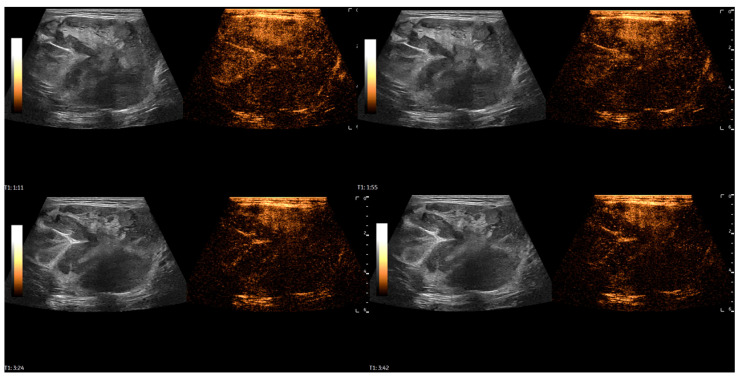
A series of CEUS images of MLS demonstrates progressive washout of the contrast agent. The total scan time was 3:42 min.

After the initial assessment, a contrast-enhanced ultrasound (CEUS) examination was performed to differentiate the mass. A high-frequency linear probe was used with a mechanical index set at 0.08. After contrast media injection (4.5 mL of SonoVue, Bracco, followed by a 5 mL flush of saline solution), scanning was performed in very short intervals, and the mass demonstrated rapid, heterogeneous enhancement, with avascular zones within the first 30 s of evaluation, followed by progressive, rapid washout of contrast media starting around 50 s. The total scan time was 3:42 min. It raised suspicion of a malignant soft-tissue mass (Figure 3 and Figure 4).

**Figure 5:**
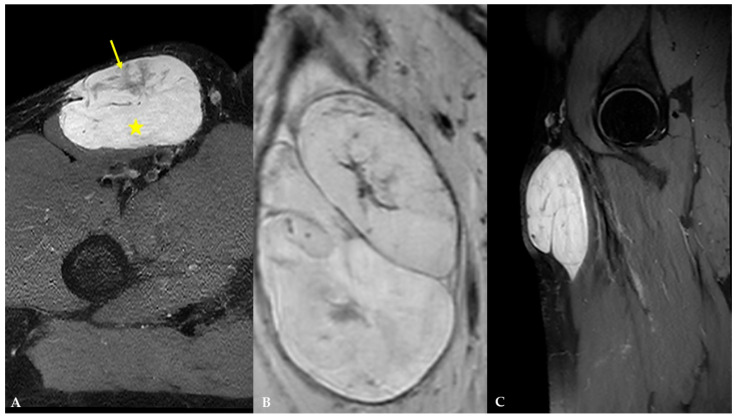
MRI features of MLS in the different planes: (**A**) axial plane, proton density (PD) fast spin echo (FSE) sequence with fat saturation demonstrating a hyperintense, lobulated, well-marginated, encapsulated mass with internal, irregular, low- to intermediate-signal-intensity foci and septa representing a mass with a high proportion of myxoid component (star) and low proportion of non-fatty non-myxoid component (arrow); (**B**) coronal plane; T2W FSE-MX 3D sequence; (**C**) sagittal plane, PD FSE sequence with fat saturation.

It was decided to perform an MRI examination (1.5 T uMR 570, Shanghai United Imaging Healthcare Co., Ltd., Shanghai, China) for more detailed anatomical assessment and preoperative planning, and to refer the patient to the tertiary sarcoma center. MRI demonstrated a lobulated, well-marginated, encapsulated mass measuring 7.2 × 4.6 × 10.6 cm, below the deep fascia, displacing the sartorius muscle without any signs of infiltration. It was isointense to adjacent muscles on T1-weighted (T1W) images and heterogeneously hyperintense on T2-weighted (T2W) images, with internal, irregular, low- to intermediate-signal-intensity foci and septa (Figure 5).

**Figure 6:**
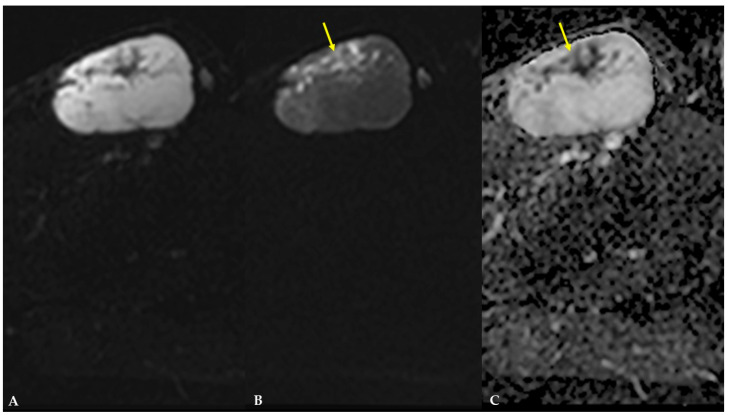
DWI and ADC images of MLS demonstrating signs of restricted diffusion within the mass (arrow): (**A**) b value = 0; (**B**) b value = 1000; (**C**) ADC map with measured ADC value of 0.8 × 10^−3^ mm^2^/s within the non-fatty non-myxoid portion and 2.5 × 10^−3^ mm^2^/s within the myxoid portion of the mass (not shown on image).

**Figure 7:**
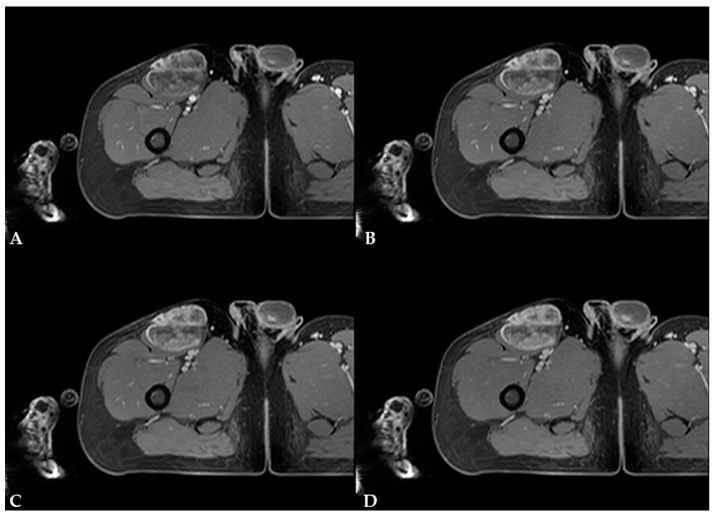
T1W DCE images showing progressive, heterogeneous enhancement of MLS, primarily involving the septa and the cellular, non-fatty, non-myxoid portion of the mass: (**A**) 1 min after contrast agent administration; (**B**) 2 min; (**C**) 3 min, and (**D**) 4 min.

On diffusion-weighted images (DWIs) and apparent diffusion coefficient (ADC) map, the previously mentioned septa and cellular portion of the mass showed restricted diffusion (Figure 6). On T1W dynamic contrast-enhanced (DCE) sequences, the mass demonstrated intense, progressive, heterogeneous opacification, primarily involving the septa and the cellular, non-fatty, non-myxoid portion of the mass (Figure 7). There were no signs of infiltration into adjacent vessels, soft-tissue, or bone structures. The proposed diagnosis was myxoid liposarcoma.

**Figure 8:**
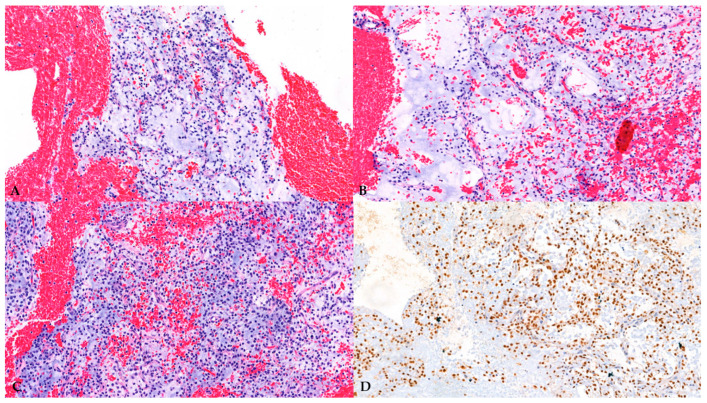
Histological examination of the biopsy specimen (**A**) H&E, magnification 2×; (**B**) H&E, magnification 10×; (**C**) H&E, magnification 10×; (**D**) immunohistochemistry (IHC), magnification 10×. It demonstrated a hypocellular tumor composed of bland, round-to-stellate cells, evenly distributed within an abundant myxoid stroma, with delicate, arborizing vasculature. No high-grade areas of the tumor were identified. IHC analysis showed diffuse positive DDIT3 staining.

Following imaging workup, at the tertiary sarcoma center, a US-guided biopsy was performed, and pathohistological diagnosis was low-grade myxoid liposarcoma (Figure 8).

**Figure 9:**
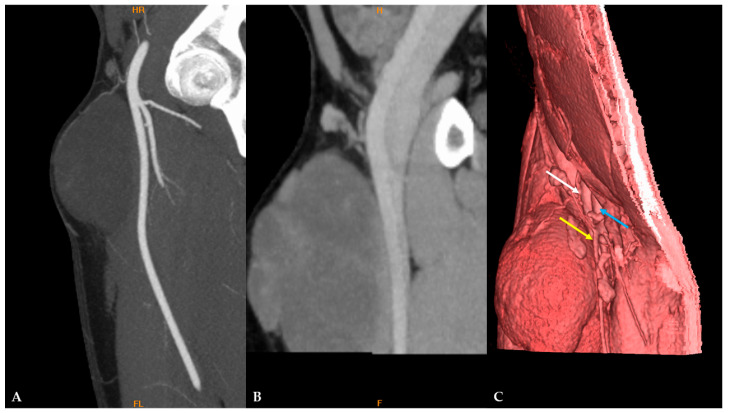
CT angiography reconstructions show the relationships between the MLS and adjacent femoral arteries and veins: (**A**,**B**) no signs of infiltration of CFA, SFA, and deep femoral veins; (**C**) 3D image with highlighted vascular structures adjacent to MLS. CFA (white arrow), deep femoral vein (blue arrow), and great saphenous vein (yellow arrow).

Contrast-enhanced computed tomography (CT) evaluation of the thorax, abdomen, and pelvis showed no specific signs of dissemination. In preparation for reconstructive surgery, CT angiography was performed, which showed that the mass was in close contact with the proximal portion of the superficial femoral artery (SFA) with no signs of infiltration. There were also no signs of infiltration of the common femoral artery (CFA) or deep femoral veins (Figure 9).

**Figure 10:**
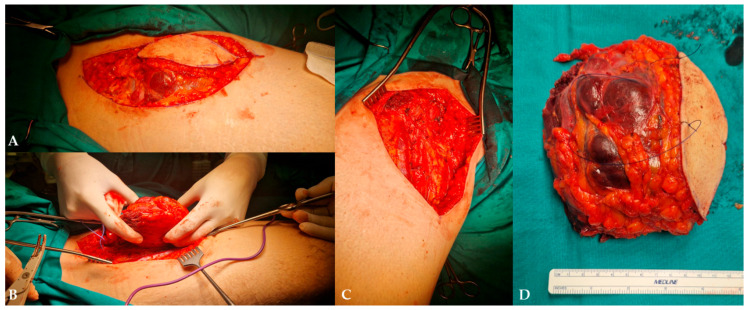
Intraoperative images of MLS: (**A**) elliptic incision, dissection, and exposure of the mass; (**B**) extirpation of the mass; (**C**) residual defect after mass removal; (**D**) macroscopic specimen for pathohistological analysis.

Plastic surgery and vascular surgery specialists performed the extirpation of the mass with the partial resection of the adjacent sartorius muscle and the complete resection of the great saphenous vein (Figure 10).

Subsequent pathohistological analysis of the mass and local lymph nodes showed clear surgical margins with no necrotic areas within the mass and no lymphatic or vascular invasion. The patient was discharged from the hospital in good general condition and is currently under regular surveillance by an oncology specialist, preparing for adjuvant radiotherapy due to the large mass size and to reduce the likelihood of local recurrence.

## 3. Discussion

MLS is the second-most common subtype of liposarcoma, often occurring in the lower limbs of patients aged 40 to 50 with no gender predilection [1,2,9]. Our patient was 38 years old at the time of the diagnosis, with the mass slowly growing over 15 years. Clinical features that often raise concern for malignancy in soft-tissue tumors include a size exceeding 5 cm, deeper location, pain, and rapid growth. However, slow-growing tumors cannot be considered benign [4]. Furthermore, if a previously stable mass or recently detected mass rapidly increases in size, both are suspicious for malignancy [4]. Therefore, in such cases, the National Institute for Health and Care Excellence (NICE) guidelines recommend ultrasound evaluation within two weeks of presentation to exclude sarcoma [4]. It is essential to determine the mass’s location relative to the muscle fascia, adjacent neurovascular structures, joints, and tendons [6].

On B-mode US, MLS usually appears as a well-defined heterogeneous mass with larger fat regions appearing as hypoechoic foci, while on Doppler evaluation, it tends to be vascular [2]. In a pictorial essay by Yang et al. [10], the authors reported MLS as a well-defined hypoechoic mass with tiny cystic foci on B-mode US. If a mass is too large to be fully imaged or measured in a single field of view, panoramic or extended field-of-view scans can be particularly useful [5]. According to a review by Griffith [6], malignant soft-tissue tumors on Doppler evaluation tend to be highly vascular, with a chaotic vascular pattern and a higher mean systolic velocity (55 cm/s) than benign tumors (27 cm/s). Also, a chaotic vascular pattern and mean systolic velocity > 50 cm/s within the tumor have 90% sensitivity and 91% specificity in differentiating benign from malignant tumors. On B-mode, the patient’s MLS showed features similar to those mentioned earlier, whereas on color and power Doppler evaluation, it was poorly vascularized, with a peak arterial systolic velocity of 13 cm/s. To improve spatial resolution and visualization of low-flow velocity arteries and veins, B-flow imaging was used, which demonstrated that MLS was moderately vascularized. Microvascular imaging (MVI) can also be used to improve visualization of low velocity flow vessels [6].

In this patient, strain elastography of MLS was performed, showing a predominantly soft mass, consistent with an elastography score of 1 according to Griffith [6]. Strain ratio and E/B (elastography/B-mode size) ratio were not measured due to technical limitations, and 2D SWE was unavailable at the time of the examination. In a case report by Lee et al. [11], breast MLS on 2D-SWE was homogenously soft with elasticity values ranging from 0 to 36 kPa. 2D SWE is less operator-dependent than strain elastography and provides both qualitative and quantitative data [6]. In Griffith’s review [6], elastography is generally described as a helpful addition to the classic US examination; however, it is not specific enough to be used solely for soft-tissue mass characterization or for differentiating benign from malignant masses. In strain elastography, adding quantitative measurements, such as the E/B ratio, may improve differentiation between benign (E/B < 1.0) and malignant tumors (E/B > 1.0) [6].

CEUS examination was performed to differentiate the patient’s mass. After contrast media injection, MLS demonstrated rapid, heterogeneous enhancement with avascular zones, followed by progressive washout of contrast agent. In their research paper from 2012, Loizides et al. [7] presented four CEUS perfusion patterns for soft-tissue masses: P1-non-enhancing mass or only rim-enhancement of the surrounding pseudo-capsule; P2-peripherally enhancing mass with non-enhancing central area; P3-diffusely enhancing mass with scattered non-enhancing regions and/or enhancement bridges; and P4-completely homogeneously enhancing masses. P1 and P4 patterns of perfusion can be considered benign, whereas the P3 pattern can be regarded as malignant. Of 54 patients, one had MLS and showed a P3 perfusion pattern on CEUS. According to study by De Marchi et al. [8] from 2015, there are seven CEUS perfusion patterns: P1-absence of contrast uptake; P2-enhancement only in the peripheral area of the lesion; P3-thin (<2 mm) and few vessels (<5/field); P4-thinner (>2 mm) and more numerous vessels (>5/field); P5-enhancement with a reticular aspect, and both thick and thin bands inside; P6-numerous vessels, important and heterogeneous enhancement with avascular areas; P7-numerous vessels in all regions, with homogeneous distribution. P6 pattern of perfusion and a very rapid perfusion (<11 s) were the most frequently observed in malignant masses. P1 and P2 of Demarchi et al. resemble P1 and P2 of Loizides et al., while P6 and P7 of Demarchi et al. resemble P3 and P4 of Loizides et al. In our case, due to visual similarity, MLS demonstrated a P3 [7] or P6 [8] perfusion pattern, with microbubbles appearing around 15 s into the scan. According to a review by Griffith [6], CEUS achieves only moderate success in differentiating benign from malignant soft-tissue tumors, with a pooled sensitivity of 76% and specificity of 67%. However, CEUS provides valuable information to improve the characterization of indeterminate soft-tissue tumors on conventional US [12]. CEUS can also help detect viable tissue for biopsy in more necrotic soft-tissue tumors [6].

MRI is the mainstay of preoperative evaluation of MLS due to its high spatial resolution, depiction of tumor extension, and depiction of tumor relationships to adjacent structures [5]. An MRI is recommended before the biopsy [5]. On MRI, MLS is usually a multilobulated, well-marginated, and encapsulated mass with four components that can be identified: a fatty component, a myxoid component, a contrast-enhancing non-fatty, non-myxoid component (or round cell tissue), and necrotic areas [1,2,9]. In the study by Löwenthal et al. [1], the authors reported that the fatty component displays high signal intensity on non-saturated T1W images, the myxoid component displays high signal intensity on T2W and low signal intensity on T1W images, the non-fatty non-myxoid component is hypointense on T1W images, displays intermediate signal intensity on T2W images, and shows contrast enhancement. At the same time, necrotic areas demonstrate low signal intensity on T1W images, low-to-intermediate signal intensity on T2W images, and no contrast enhancement. In this case, MLS presented as a lobulated, well-marginated, encapsulated mass with an isointensity to adjacent muscles on T1W images and heterogeneously high signal intensity on T2W images, with internal, irregular, low- to intermediate-signal-intensity foci and septa indicating that it is composed of a high proportion of myxoid component and a low proportion of non-fatty non-myxoid component. When attempting to differentiate between low-grade and high-grade MLS on MRI, Löwenthal et al. [1] have found that low-grade MLS were smaller, had a pseudo-capsule, and had a higher proportion of fatty and myxoid components. In contrast, high-grade MLS had a higher proportion of a non-fatty non-myxoid component and tumor necrosis. No typical contrast enhancement was identified; however, heterogeneous enhancement was the most encountered pattern in both low and high-grade MLS. High-grade MLS also showed signs of circular encasement of large vessels and bone infiltration. In this case, MLS demonstrated intense, progressive, heterogeneous enhancement, primarily involving the septa and the cellular, non-fatty, non-myxoid portion of the mass. In their review, Scalas et al. [5] also noted that signal heterogeneity on T1W and T2W images is more commonly observed in high-grade MLS, which is associated with a poorer prognosis. In another study by Gruber et al. [13], the authors reported that, according to Loizides et al. [7], P2 and P3 patterns of contrast enhancement on MRI were strong predictors of malignancy in soft-tissue masses. In our case, the patient had a low-grade MLS with abundant myxoid component and heterogeneous contrast enhancement. According to Sung et al. [9], MLS with heterogeneous enhancement consists of two distinct zones. Compact cellularity, a prominent capillary network, and a myxoid pattern characterize the enhancing zone. In contrast, the non-enhancing zone is characterized by necrosis with or without hemorrhage, mucinous material, and a less cellular myxoid portion without capillary networks. In the study by Encinas Tobajas et al. [3], 83.3% of MLS with heterogeneous enhancement were high-grade. The ADC values obtained for MLS ranged from 1.1 to 2.5 × 10^−3^ mm^2^/s, with a median of 2.0 × 10^−3^ mm^2^/s, and showed no statistical difference between high-grade and low-grade subtypes. The ADC values in MLS in this case were 0.8 × 10^−3^ mm^2^/s within the non-fatty non-myxoid portion and 2.5 × 10^−3^ mm^2^/s within the myxoid portion of the mass. According to a study by Hua et al. [14], mean ADC values of the non-fatty non-myxoid region in the good prognosis group were 1.66  ±  0.23 × 10^−3^ mm^2^/s, whereas the corresponding values in the poor prognosis group were 1.21  ±  0.41 × 10^−3^ mm^2^/s. 

Suspicious or likely malignant soft-tissue tumors should undergo biopsy, which is the gold standard for diagnosis; however, the patient should be referred to a sarcoma reference center before biopsy [15]. Percutaneous biopsy is often performed under US guidance to improve safety [6]. CEUS can also help identify areas of viable tumors for targeted biopsy [6]. In this case, the biopsy sample was sent for molecular analysis, but due to suboptimal sample quality, the analysis was insufficient and uninterpretable. According to the WHO’s classification of Soft-Tissue Tumors [16], essential criteria for diagnosis of myxoid liposarcoma are characteristic histology (myxoid matrix containing delicately arborizing capillaries; bland round to ovoid cells; variable number of small non-pleomorphic lipoblasts, often adjacent to capillaries), whereas molecular analysis is a desirable criterion, but not essential. In this case, given the appropriate clinical context, the patient’s age, characteristic histology, and diffuse nuclear positivity for DDIT3 immunostain, a highly sensitive and specific marker for the diagnosis, the diagnosis of myxoid liposarcoma was made with high certainty.

In the differential diagnosis, soft-tissue hematoma, extraskeletal myxoid chondrosarcoma, intramuscular myxoma, ganglion, and myxoid malignant fibrous histiocytoma must be considered [4,9]. In this case, the patient denied any trauma or recent surgery of the right femoral region, and based on imaging findings, soft-tissue hematoma was excluded. Differentiation of intramuscular myxoma from MLS can be challenging; it has been differentiated based on MLS showing intense enhancement (65–100% of tumor volume) [9]. Ganglion was excluded because of its cystic morphology, and it typically does not show contrast enhancement [9]. Other soft-tissue tumors have been excluded based on histopathological findings.

MLS shows atypical metastatic spread with a high proportion of extrapulmonary metastases (paraspinal soft-tissues, retroperitoneum, and spine) [2,17]. According to the European Society of Musculoskeletal Radiology’s recent guidelines [17], for MLS, due to low PET FDG avidity, whole-body MRI is strongly recommended for the detection of bone and extraskeletal metastases and for staging. Furthermore, MLS is highly sensitive to chemo- and radiotherapy [18]. The limitation in this case is that the patient underwent CT staging of the thorax, abdomen, and pelvis rather than a whole-body MRI due to technical reasons (specific MRI protocol in development). Nevertheless, the patient is under regular surveillance by an oncology specialist and is preparing for adjuvant radiotherapy due to the large mass size and to reduce the likelihood of local recurrence.

## 4. Conclusions

MLS is a malignant soft-tissue tumor and the second-most common subtype of liposarcoma, often occurring in the lower limbs of middle-aged patients. They usually present with a slow-growing, soft, painless mass. MPUS is the first imaging modality, and MRI is the mainstay for imaging assessment. According to new guidelines, whole-body MRI is strongly recommended for detecting bone and extraskeletal metastases and for staging. A multidisciplinary approach is essential in the management of patients with MLS because it provides a tailored, individualized assessment from diagnosis through treatment, helping ensure the best possible outcome.

## Data Availability

Data is contained within the article, and further inquiries can be directed to the corresponding author.

## References

[1] Löwenthal D., Zeile M., Niederhagen M., Fehlberg S., Schnapauff D., Pink D., Tunn P.U., Reichardt P., Hamm B., Dudeck O. (2014). Differentiation of myxoid liposarcoma by magnetic resonance imaging: A histopathologic correlation. Acta Radiol..

[2] de Boer H.C., Musson R. (2023). Imaging features of myxoid soft-tissue tumours. Clin. Radiol..

[3] Encinas Tobajas V.M., Almeida González C., Marcilla D., Vallejo M., Cano Rodríguez A., Reina Sánchez de Movellán J.I., Morales Pérez J.M. (2023). Myxoid liposarcoma: MRI features with histological correlation. Radiologia.

[4] Berry C., Charnock M. (2025). Sarcoma or haematoma? If only it was that simple! Part 1. Ultrasound.

[5] Scalas G., Parmeggiani A., Martella C., Tuzzato G., Bianchi G., Facchini G., Clinca R., Spinnato P. (2021). Magnetic resonance imaging of soft tissue sarcoma: Features related to prognosis. Eur. J. Orthop. Surg. Traumatol..

[6] Griffith J.F. (2023). Practical approach to ultrasound of soft tissue tumors and the added value of MRI: How I do it. J. Ultrason..

[7] Loizides A., Peer S., Plaikner M., Djurdjevic T., Gruber H. (2012). Perfusion pattern of musculoskeletal masses using contrast-enhanced ultrasound: A helpful tool for characterisation?. Eur. Radiol..

[8] De Marchi A., Prever E.B.D., Cavallo F., Pozza S., Linari A., Lombardo P., Comandone A., Piana R., Faletti C. (2015). Perfusion pattern and time of vascularisation with CEUS increase accuracy in differentiating between benign and malignant tumours in 216 musculoskeletal soft tissue masses. Eur. J. Radiol..

[9] Sung M.S., Kang H.S., Suh J.S., Lee J.H., Park J.M., Kim J.Y., Lee H.G. (2000). Myxoid liposarcoma: Appearance at MR imaging with histologic correlation. Radiographics.

[10] Yang D.M., Kim H.C., Kim S.W., Won K.Y. (2020). Groin abnormalities: Ultrasonographic and clinical findings. Ultrasonography.

[11] Lee S.J., Ryu J.K., Won K.Y., Han S.A. (2023). Myxoid Liposarcoma of the Breast Mimicking Phyllodes Tumor: A Case Report. J. Korean Soc. Radiol..

[12] Hu Y., Li A., Wu M.J., Ma Q., Mao C.L., Peng X.J., Ye X.H., Liu B.J., Xu H.X. (2023). Added value of contrast-enhanced ultrasound to conventional ultrasound for characterization of indeterminate soft-tissue tumors. Br. J. Radiol..

[13] Gruber L., Loizides A., Luger A.K., Glodny B., Moser P., Henninger B., Gruber H. (2017). Soft-Tissue Tumor Contrast Enhancement Patterns: Diagnostic Value and Comparison Between Ultrasound and MRI. AJR Am. J. Roentgenol..

[14] Hua J., Yang W., Li A., Wang S., Ying M. (2025). Magnetic resonance imaging assessing the correlation of components and prognosis in myxoid liposarcoma. Acta Radiol..

[15] Noebauer-Huhmann I.M., Vanhoenacker F.M., Vilanova J.C., Tagliafico A.S., Weber M.A., Lalam R.K., Grieser T., Nikodinovska V.V., de Rooy J.W.J., Papakonstantinou O. (2024). Soft tissue tumor imaging in adults: European Society of Musculoskeletal Radiology-Guidelines 2023-overview, and primary local imaging: How and where?. Eur. Radiol..

[16] Antonescu C.R., Blay J.-Y., Bovée J.V.M.G., Bridge J.A., Cunha I.W., Dei Tos A.P., Flanagan A.M., Fletcher C.D.M., Folpe A.L., Gronchi A. (2020). WHO Classification of Tumours: Soft Tissue and Bone Tumours.

[17] Noebauer-Huhmann I.M., Vanhoenacker F.M., Vilanova J.C., Tagliafico A.S., Weber M.A., Lalam R.K., Grieser T., Nikodinovska V.V., de Rooy J.W.J., Papakonstantinou O. (2025). Soft tissue tumor imaging in adults: Whole-body staging in sarcoma, non-malignant entities requiring special algorithms, pitfalls and special imaging aspects. Guidelines 2024 from the European Society of Musculoskeletal Radiology (ESSR). Eur. Radiol..

[18] Machałowski T., Gutowski P., Zagrodnik E., Godlewska A., Śmieja K., Kawałek O., Grzymała-Figura A., Ciećwież S.M., Gross-Kępińska K., Szczuko M. (2025). Myxoid Liposarcoma of the Thigh in Early Puerperium—Rare Case Report and Narrative Review. Diseases.

